# Persistent-relapsing SARS-CoV-2 infection following rituximab treatment for autoimmune rheumatic diseases: diagnosis and outcomes

**DOI:** 10.1136/rmdopen-2025-005756

**Published:** 2025-07-21

**Authors:** Katerina Chavatza, Elisavet Mastrostamati, Charalampos Charalampidis, Elvira-Markela Antonogiannaki, Ioannis Grigoropoulos, Emmanouil Karofylakis, Foteini Gkolemi, Georgios Koromvokis, Electra Kalara, Eleni Sambatakaki, Antonis Fanouriakis, Konstantinos Thomas

**Affiliations:** 1 Rheumatology-Clinical Immunology Unit, 4th Department of Internal Medicine, Attikon University General Hospital, National and Kapodistrian University of Athens, Athens, Greece; 2 4th Department of Internal Medicine, Attikon University General Hospital, National and Kapodistrian University of Athens Medical School, Athens, Greece; 3 2nd Department of Pulmonary Medicine, Attikon University General Hospital, National and Kapodistrian University of Athens Medical School, Athens, Greece

**Keywords:** Infections, Rituximab, COVID-19

## Abstract

**Background:**

COVID-19 may persist or relapse in patients on B-cell depleting biologic therapies.

**Objective:**

To examine the rate and outcome of persistent-relapsing COVID-19 (prCOVID-19) in patients with autoimmune rheumatic diseases (AIRD) treated with rituximab (RTX).

**Methods:**

Single-centre, retrospective cohort study of patients diagnosed with prCOVID-19 (June 2021 to January 2025). prCOVID-19 was defined as persistence of symptoms and lung imaging findings for >30 days, along with persistently positive or PCR-based conversion in upper or lower respiratory tract samples.

**Results:**

26 out of 225 (11.6%) AIRD patients, previously diagnosed with COVID-19 during RTX treatment period, developed 27 prCOVID-19 events (females: 20 (76.9%), median age: 61 years, median disease duration: 5.5 years, ≥3 COVID-19 vaccine doses: 20 (76.9%)). No prCOVID-19 infection in a control sample of 661 patients treated with other biologic/targeted synthetic/conventional synthetic disease-modifying antirheumatic drugs was documented. Median cumulative RTX dose was 12 g, while in 17 (68%) prCOVID-19 events, IgG levels were below 700 mg/L. Median duration of prCOVID-19 infection was 65 (IQR 74) days and median duration of hospitalisation 10.5 (IQR 14) days. 11 patients (42.3%) had ≥2 hospitalisations, 3 patients needed mechanical ventilation and 4 deaths were recorded. 59 of 113 (52.2%) nasopharyngeal PCR samples (NPS) and 12/17 (70.6%) bronchoalveolar lavage (BAL) PCR samples were positive during prCOVID-19. Bronchoscopy established the diagnosis of prCOVID-19 in 33% of events.

**Conclusion:**

AIRD patients treated with RTX are at risk for prCOVID-19. In such patients, the diagnostic accuracy of NPS PCR is suboptimal, necessitating PCR testing in BAL when prCOVID-19 is highly suspected.

**WHAT IS ALREADY KNOWN ON THIS TOPIC**

- Rituximab (RTX) has been associated with reduced efficacy of SARS-CoV-2 vaccination and more severe COVID-19 outcomes in patients with autoimmune rheumatic diseases (AIRD).
- The frequency and disease course of persistent or relapsing COVID-19 (prCOVID-19) in these patients is unknown.

**WHAT THIS STUDY ADDS**

- prCOVID-19 disease in RTX-treated AIRD patients leads to longer disease duration and worse outcomes.
- A high percentage of negative nasopharyngeal PCR tests was found in patients with prCOVID-19, necessitating the use of PCR in bronchoalveolar lavage (BAL) to establish the diagnosis.

**HOW THIS STUDY MIGHT AFFECT RESEARCH, PRACTICE OR POLICY**

- In patients with AIRD treated with RTX, an aggressive work-up, including BAL PCR, is needed if prCOVID-19 is suspected.
- Empirical antiviral therapy should also be considered in patients with a high clinical suspicion for prCOVID-19.

## Introduction

 Despite the declaration of the end of the COVID-19 pandemic by WHO in May 2023, immunocompromised patients are still disproportionately affected by SARS-CoV-2.^1 2^ Rituximab (RTX) is an anti-CD20 monoclonal antibody indicated for haematological malignancies and autoimmune rheumatic diseases (AIRD). RTX-treated patients are at higher risk for adverse COVID-19 outcomes.^3 4^ A pattern of persistent/relapsing infection has been recognised from the early phases in RTX-treated patients.^5^ In these patients, the virus may reside within tissue reservoirs and its ongoing replication may lead to new, divergent variants that escape established immunity.^6 7^ However, after vaccination, boosted spike-specific memory CD4 T cells on SARS-CoV-2 booster vaccination may compensate, at least in part, for iatrogenic B-cell depletion.^8 9^ RTX dose reduction or interruption has been raised, especially for patients with limited alternative therapeutic options.^10^

In this study, we examined the frequency and outcome of persistent or relapsing COVID-19 (prCOVID-19) in a large cohort of RTX-treated AIRD patients.

## Methods

### Study design

This was a single-centre, retrospective cohort study from Attikon University Hospital, Athens, Greece, including patients with AIRD who received RΤΧ between June 2021 and January 2025 and were diagnosed with prCOVID-19. For control, we scrutinised our patient cohort for prCOVID-19 cases in patients treated with other biologic (biologic or targeted synthetic disease-modifying antirheumatic drugs (b/tsDMARDs)) or synthetic antirheumatic drugs (conventional synthetic disease-modifying antirheumatic drugs (csDMARDs)).

### Definitions

PrCOVID-19 was defined as a combination of the following: (1) ongoing respiratory and/or systemic symptoms for >30 days, with one of the following patterns: (i) chronic pattern (*persistent*), that is, ongoing or progressive respiratory symptoms, systemic symptoms or both; or (ii) relapsing disease, with asymptomatic or pauci-symptomatic periods, followed by episodes of symptomatic COVID-19;^7^ (2) unilateral or bilateral pulmonary opacities demonstrated on chest CT imaging and (3) PCR identification of SARS-CoV-2 in an upper (nasopharyngeal, NPS) or lower bronchoalveolar lavage (BAL) respiratory tract sample.^11^

PrCOVID-19 was considered resolved with the end of patient symptoms, without need for laboratory testing. PCR testing was implemented in NPS and/or BAL samples to establish the diagnosis. The decision to perform bronchoscopy and PCR testing in BAL was taken on clinical grounds. We assessed concordance rates between recent NPS PCR and the BAL PCR (NPS+/BAL− or NPS−/BAL+) in patients who underwent both procedures. An NPS sample was considered recent if performed within 45 days before BAL, during the symptomatic period.^12^

Chest CT findings were classified as ground-glass opacities and/or consolidations. Severe prCOVID-19 was defined as respiratory failure requiring supplemental oxygen via Venturi mask (at least 31%), high-flow nasal cannula or mechanical ventilation. Hypogammaglobulinaemia was defined as IgG<700 mg/L.

Demographics, disease characteristics and current treatments, COVID-19 clinical and laboratory features and associated therapies and outcomes, were retrieved from chart review, electronic medical records and patient interviews. Data regarding vaccination status and results of NPS/BAL tests were collected from the National COVID-19 Registry.

### Statistics

Median (IQR) values and frequencies (%) were used for continuous and categorical variables, respectively. Swimmer plot of disease course and PCR results was constructed using R Statistical Software (V.4.3.3; R Core Team 2024), ‘ggplot’ library and ‘ggnewscale’ R package. Mann-Whitney-Wilcoxon test and χ^2^ test were used for comparisons of clinically important variables with outcomes.

## Results

### Baseline clinical and imaging characteristics

Among the 470 patients who received RTX during the study period, 225 experienced COVID-19 at least once. Of them, 26 (11.6%) developed 27 prCOVID-19 events; a single patient developed prCOVID two times, with a time lag of 15 months between the two events. From the control group (661 patients), 45.4% received b/tsDMARDs and 54.6% csDMARDs. None of them developed prCOVID-19. 20 (77%) patients were fully vaccinated (table 1). Nine (35%) patients had ANCA-associated vasculitis, eight (31%) rheumatoid arthritis, six (23%) systemic lupus erythematosus and three (12%) inflammatory myopathies. 14 patients (54%) had current or past lung involvement. The median (IQR) duration of the rheumatic disease was 5.5 (4.8) years, while the cumulative RTX dose prior to prCOVID-19 diagnosis was 12 (6.5) g. Among the 25 events with available data, 17 had low IgG levels (median 550 mg/L, IQR 367). All the events had ground glass infiltrates in chest CT, while consolidations were observed in 16 (59.3%).

**Table 1:** Patient characteristics (n=26)

Variable	Results
Female, n (%)	20 (76.9)
Male, n (%)	6 (23.1)
Age, years, median (IQR)	61 (19)
Time interval between last rituximab and SARS-CoV-2 disease, months, median (IQR)	3.6 (3.8, max 11.4)
Duration of hospitalisation, days, median (IQR)	10.5 (14, max 70)
Previous SARS-CoV-2 vaccine doses, n (%)	
0	4 (15.4)
1	0 (0)
2	2 (7.7)
3	13 (50)
≥4	7 (26.9)
Low-dose glucocorticoids, n (%)*	20 (76.9)
Comorbidities, n (%)	20 (80.8)
Need for hospitalisation, n′ (%)*	26 (96.3)
Outcome, n (%)* Discharge	22 (84.6)

* Low-dose steroids: prednisone ≤5 mg. From the rest of the patients, none of them received prednisone higher than 5 mg at the time of diagnosis.

n, number of prCOVID-19 patients; n', number of persistent or relapsing COVID-19 (prCOVID-19) events; n'', prCOVID-19 events with need of hospitalisation.

### Diagnosis of prCOVID-19

#### Initial COVID-19 diagnosis

In eight prCOVID-19 events (29.6%), initial COVID-19 infection was diagnosed with a rapid antigen test (RAT), 17 (63%) with NPS test, 1 with a BAL PCR and 1 with a PCR test of bronchial secretions during intubation (table 2). Median (IQR) time to diagnosis with either RAT or any PCR test was 2 (12.5) days (maximum 92 days), while median (IQR) time to PCR test confirmation was 14 (29.5) days (maximum 107). In nine events with a relapsing pattern (concerning relapses that led to hospitalisation), a diagnostic delay of the relapse was recorded, and median (IQR) time to diagnosis was 8 (IQR 15) days (maximum 90 days).

**Table 2:** Patients’ detailed characteristics

No.	Sex	Age	Diagnosis	Vaccine doses (n)	Month/year of diagnosis	Symptoms duration until diagnosis (weeks)*	Lung involvement of underlying AIRD	Days of hospitalisation (Hospitalisations (n))	ICU admission	Antivirals	Intravenous immunoglobulin	Evidence of coinfection(s)	COVID-19 outcome
1	F	73	GPA*	2	February/2023	4 (RAT*)	Yes	10 (1)	No	Remdesivir	No	No	Recovery
2	M	57	Antisynthetase S.	3	February/2022	1 (PCR-NPS)	Yes	43 (1)	No	Remdesivir	No	No	Recovery
3	F	39	SLE*/Sjogren	3	November/2023	14 (PCR-BAL)	No	18 (2)	No	Remdesivir Nirmatrelvir/Ritonavir	Yes	*H. influenzae*	Recovery
4	F	63	GPA	4	May/2023	1 (PCR-NPS)	Yes	10 (1)	No	Remdesivir	No	No	Recovery
5	F	58	SLE/MS*	4	November/2022	1 (PCR-NPS)	No	60 (2)	Yes	Remdesivir Nirmatrelvir/Ritonavir	No	No	Recovery
6A	F	74	Dermatomyositis	3	June/2022	1 (PCR-NPS)	Yes	13 (2)	No	Remdesivir	NA*	*H. influenzae*	Recovery
6B	F	75	Dermatomyositis	3	September/2023	3 (PCR-NPS)	Yes	45 (2)	No	Remdesivir Nirmatrelvir/Ritonavir	Yes	No	Recovery
7	F	58	SLE	3	February/2023	1 (PCR-NPS)	No	15 (1)	No	Remdesivir Nirmatrelvir/Ritonavir	No	No	Recovery
8	F	33	SLE/MS	0	October/2022	1 (RAT)	No	31 (2)	No	Remdesivir	NA	No	Recovery
9	F	24	GPA	3	June/2022	2 (PCR-NPS)	Yes	40 (5)	No	Remdesivir Nirmatrelvir/Ritonavir	Yes	*H. influenzae*	Recovery
10	F	62	RA-ILD*	4	August/2024	5 (PCR-NPS)	Yes	26 (2)	No	Remdesivir Nirmatrelvir/Ritonavir	Yes	No	Recovery
11	F	61	RA	3	August/2022	1 (PCR-NPS)	No	61 (1)	Yes	Remdesivir	NA	No	Death
12	F	60	GPA	3	November/2023	13 (PCR-tracheal aspiration)	Yes	48 (2)	Yes	Remdesivir	Yes	No	Recovery
13	F	75	Dermatomyositis	3	September/2022	2 (PCR-NPS)	Yes	22 (1)	No	Remdesivir	NA	No	Recovery
14	M	87	GPA	3	November/2023	3 (PCR-NPS)	Yes	23 (1)	No	Remdesivir	NA	No	Death
15	M	78	GPA	4	September/2023	1 (RAT)	Yes	19 (1)	No	Remdesivir Nirmatrelvir/Ritonavir	Yes	*Serratia*, *S. aureus*	Recovery
16	M	52	EGPA*	3	September/2023	1 (RAT)	No	11 (1)	No	Remdesivir	Yes	No	Recovery
17	F	74	GPA	3	August/2023	1 (RAT)	Yes	9 (1)	No	Remdesivir Nirmatrelvir/Ritonavir	No	No	Death
18	F	75	RA-ILD	4	October/2022	1 (RAT)	No	25 (2)	No	Remdesivir	No	No	Recovery
19	F	76	RA	4	July/2023	1 (PCR)	No	70 (2)	No	Remdesivir Nirmatrelvir/Ritonavir	No	No	Recovery
20	F	62	RA/Scleroderma	2	September/2023	1 (RAT)	No	10 (1)	No	Remdesivir Nirmatrelvir/Ritonavir	No	No	Recovery
21	F	51	SLE/MS	3	September/2024	1 (RAT)	No	4 (1)	No	Remdesivir	No	*H. influenzae*	Recovery
22	F	55	RA	4	May/2023	2 (PCR-NPS)	No	40 (3)	No	Remdesivir Nirmatrelvir/Ritonavir	No	No	Recovery
23	M	61	RA	0	October/2024	1 (PCR-NPS)	No	57 (1)	Yes	Remdesivir	No	No	Death
24	M	57	RA-ILD	0	January/2025	4 (PCR-NPS)	Yes	10 (1)	No	Remdesivir Nirmatrelvir/Ritonavir	Yes	No	Recovery
25	F	75	GPA	0	January/2025	1 (PCR-NPS)	Yes	29 (2)	No	Remdesivir	No	No	Recovery
26	F	47	SLE	3	August/2021	1 (PCR-NPS)	No	0 (0)	No	None	No	No	Recovery

* Symptoms duration until diagnosis (weeks): week of disease course when patient had the first positive diagnostic test after symptom onset

AIRD, autoimmune rheumatic diseases; BAL, bronchoalveolar lavage; EGPA, eosinophilic granulomatosis with polyangiitis; GPA, granulomatosis with polyangiitis; ICU, intensive care unit; MS, multiple sclerosis; NA, missing data; NPS, nasopharyngeal; PA, eosinophilic granulomatosis with polyangiitis; RA-ILD, rheumatoid arthritis-interstitial lung disease; RAT, rapid antigen test; SLE, systemic lupus erythematosus.

#### Role of bronchoscopy in prCOVID-19

**Figure 1:**
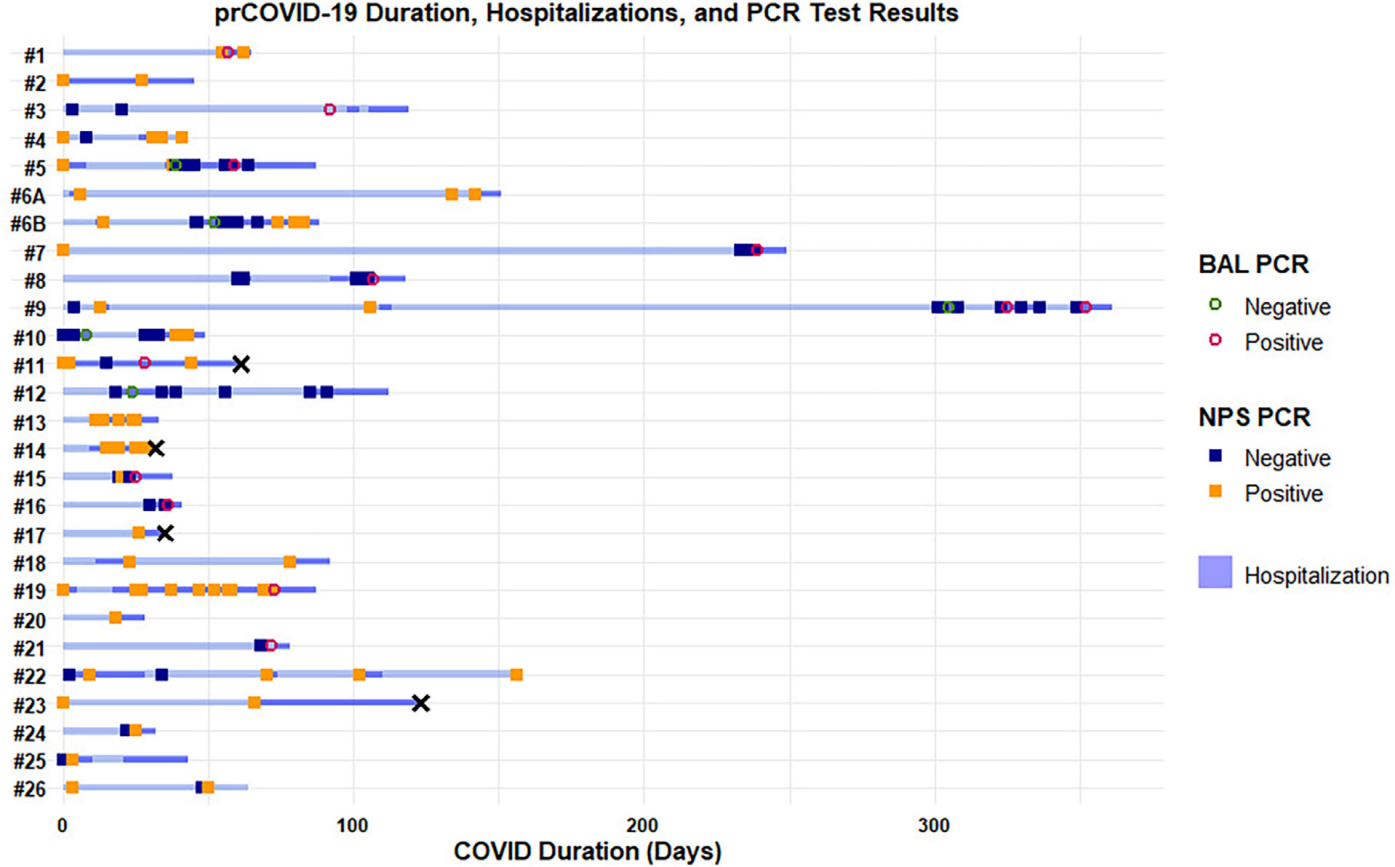
Swimmer plot of disease course. Deaths are denoted with an ‘x’. BAL, bronchoalveolar lavage; NPS, nasopharyngeal; prCOVID-19, persistent or relapsing COVID-19.

A total of 113 NPS and 17 BAL PCR tests were performed in the 27 prCOVID-19 events. Median (IQR) number of NPS PCR tests per patient was 3 (4), with a positivity rate of 52.2% (59/113). Among the 17 BAL PCR tests, 70.6% (12/17) were positive during active COVID-19. The discordance rate of BAL+/NPS− was 9/28 (32.1%) (online supplemental figure 1). In five (19%) prCOVID-19 events, a coinfection was observed (table 2). In more detail, one patient (#3, table 2 and figure 1) was diagnosed directly by BAL PCR after repeatedly negative NPS PCR. Three patients (#8, #16, #21), initially diagnosed with RAT, had the following negative NPS PCR, and the diagnosis was finally made by positive BAL PCR. Five patients (#5, #7, #9, #11, #15) had a similar pattern, as they were initially diagnosed with positive NPS PCR; nevertheless, this was followed by multiple negative NPS PCR tests, and the patients were finally diagnosed with the molecular detection of SARS-CoV-2 by PCR in BAL samples. Two patients (#1, #19) with a priorly established prCOVID-19 diagnosis underwent bronchoscopy for exclusion of coinfections. Collectively, bronchoscopy contributed to the diagnosis of prCOVID-19 in 9/27 (33%) events.

### Treatment

Eight prCOVID-19 events were treated with an antiviral agent within the first week of symptom onset. All events except one received remdesivir, while in 13 prCOVID-19 events, both remdesivir and nirmatrelvir/ritonavir were given. Eight of 22 events received intravenous immunoglobulin (IVIG) treatment during hospitalisation, while only two events did not receive antibiotics.

### Outcomes

Median (IQR) duration of prCOVID-19 was 65 (74) days, maximum 361 days, with a relapsing disease pattern in 16/27 (59.3%). The median (IQR) duration of hospitalisation was 10.5 (14) days, with 11 patients having ≥2 hospitalisations. Twelve prCOVID-19 events experienced severe respiratory failure and three needed mechanical ventilation. Four deaths (15%) were recorded. Details per patient are presented in figure 1. Results from inferential statistics are presented in online supplemental table 1.

## Discussion

We present the largest cohort of RTX-treated AIRD patients who developed prCOVID-19. Importantly, this exclusively affected RTX-treated patients, since we did not find any case of prCOVID-19 in patients treated with other treatments.

During the predominance of the less virulent Omicron variant, RTX-treated patients exhibit a lower risk for severe COVID-19, if fully vaccinated.^13^ PrCOVID-19, however, seems to affect patients regardless of the circulating variant, since all but one of our patients were diagnosed during the Omicron predominance. Of note, 46.2% developed severe respiratory failure, despite prior vaccination which may be due to the high prevalence of hypogammaglobulinaemia and other comorbidities, identified as risk factors for severe breakthrough infection.^13^ Our analysis did not identify variables associated with severe prCOVID-19.

A notable finding of our study was the suboptimal diagnostic yield of NPS PCR testing, resulting in diagnostic delay, treatment delay and therefore disease prolongation. Our overall BAL+/NPS− discordance rate was 29%, greater than the range reported in the general population (4%–25%),^12^ but in agreement with other smaller cohorts of RTX-treated haematological patients.^11^ Our study underlines the crucial role of bronchoscopy in the early diagnosis of prCOVID-19, and the diagnosis of bacterial coinfections (19% in our cohort), similar to patients with cancer (13%–17%),^12 14^ but not in the general population.^15^ Even though all the recovered co-pathogens were considered clinically important and treated, the exact significance of coinfection remains currently unknown.^12 14^

Our study highlights the uncertainty in the management of prCOVID-19. We prescribed combination antiviral therapy in 50% of the patients, a practice associated with increased rates of viral clearance in B-cell-depleted patients.^16 17^ A significant proportion of hypoglobulinaemic patients were also treated with IVIG, a potentially beneficial intervention, since recently produced commercial IVIG products contain sufficient titres of neutralising antibodies.^18^

The main strengths of our study include the relatively large sample size, compared with other similar studies in the literature,^16 17^ and more importantly, the inclusion of exclusively AIRD patients that provided a more homogeneous population. The retrospective, single-centre observational design is its main limitation, yet gave us the opportunity to gather detailed patient-level data.

In conclusion, prCOVID-19 infection is not uncommon among RTX-treated AIRD patients and usually leads to longer disease duration and worse outcomes, despite antiviral treatment. An aggressive diagnostic work-up, including BAL PCR, is needed in suspected cases with an initially negative NPS PCR test; moreover, empirical antiviral therapy should be considered in patients with a high clinical suspicion for prCOVID-19.

## Supplementary material

10.1136/rmdopen-2025-005756 online supplemental file 1
