# Supplementary material for: Persistent-relapsing SARS-CoV-2 infection following rituximab treatment for autoimmune rheumatic diseases: diagnosis and outcomes
Source: RMD Open. 2025 Jul 21;11(3):e005756. doi: 10.1136/rmdopen-2025-005756 (PMC12281322; doi:10.1136/rmdopen-2025-005756)
Supplement: online supplemental file 1 [file rmdopen-11-3-s001.pdf]

Supplementary figure 1. Discordance rate of BAL+ among NPS- (9/28=32.1%). The majority of BAL + was matched with >1 NPS test.

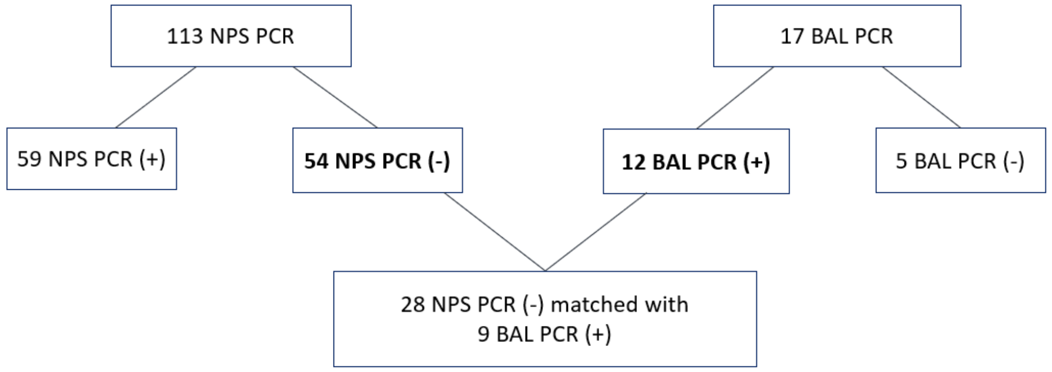

Supplementary table 1. Inferential statistics for comparisons of clinically important variables with outcomes (duration of hospitalization and disease severity).

Groups	Hospitalization duration (Mann-Whitney-Wilcoxon test)	Severe respiratory failure (Chi square test)
**Age (≥61 *vs* < 61 y)**	p value: 0.4	p value: 0.64
**Pre-existing lung disease (Yes/No)**	p value: 0.67	p value: 0.98
**Cumulative RTX dose (≥12 *vs* <12 g)**	p value: 0.64	p value: 0.73
**Disease pattern (Relapsing vs not relapsing)**	**p value: 0.03**	p value: 0.99
